# Wassermann Reaction in Cadavers

**Published:** 1913-11

**Authors:** 


WASSERMANN REACTION IN CADAVERS. Major, *Johns Hopkins Hospt. Bull.,* discuss Bruck's claim that the Wassermann reaction is purely billogic and does not occur in cadavers. He found the reaction in the serum of cadavers not showing syphilitic lesions, death being due to various diseases. Fraenkel and Much and also Nauwerck and Weichert (the latter reporting a series of 200) on the contrary, claim an agreement between antemortem and post-mortem reactions in nineteen cases, the remainder of the series of 200 showing consistent results but not checked before and after death. Schmidt found an agreement in all of thirty-two cases examined before and after death. Major found an agreement in twenty-four of twenty-five cases. The remaining case died some time after vigorous anti-syphilitic treatment and the reaction, positive on admission was negative post-mortem. Major thinks that discrepancies, aside from the result of treatment, may be explained by the fact that careful necropsy may fail to show characteristic lesions although the case is really syphilitic.

